# In-cell NMR in *E. coli* to Monitor Maturation Steps of hSOD1

**DOI:** 10.1371/journal.pone.0023561

**Published:** 2011-08-24

**Authors:** Lucia Banci, Letizia Barbieri, Ivano Bertini, Francesca Cantini, Enrico Luchinat

**Affiliations:** 1 Magnetic Resonance Center, University of Florence, Sesto Fiorentino, Italy; 2 Department of Chemistry, University of Florence, Sesto Fiorentino, Italy; Griffith University, Australia

## Abstract

In-cell NMR allows characterizing the folding state of a protein as well as posttranslational events at molecular level, in the cellular context. Here, the initial maturation steps of human copper, zinc superoxide dismutase 1 are characterized in the *E. coli* cytoplasm by in-cell NMR: from the apo protein, which is partially unfolded, to the zinc binding which causes its final quaternary structure. The protein selectively binds only one zinc ion, whereas *in vitro* also the copper site binds a non-physiological zinc ion. However, no intramolecular disulfide bridge formation occurs, nor copper uptake, suggesting the need of a specific chaperone for those purposes.

## Introduction

Folding and maturation of proteins characterized by post-translational modifications and formation of quaternary structure is a complex process which progresses through a number of well concerted events. A deep understanding of such processes requires their characterization at molecular level in a cellular context.

In-cell NMR has the unique ability to acquire structural and conformational information of biomolecules in their native cellular environment at atomic level [1], [2]. It has been previously shown that the bacterial cytoplasm is a good model of the eukaryotic one, especially to study the effects of molecular crowding on protein folding and non-specific interactions [3], as they have similar pH and redox potential [4]–[6].

Within this frame, we have characterized by in-cell NMR the wild-type human copper, zinc superoxide dismutase 1 (hSOD1) protein, as well as the initial steps towards its maturation.

hSOD1 is a 32 kDa homodimeric protein involved in the cellular defence against oxidative stress. It is physiologically expressed at relatively high concentrations in human cells, and it exerts its function in the cytoplasm, in the nucleus and in the mitochondrial IMS [7]. In order to reach its mature form, hSOD1 has to incorporate one Zn^2+^ ion and one catalytic Cu^+^ ion per subunit. Additionally, two conserved cysteine residues (Cys 57 and 146) form an intramolecular disulfide bridge during the protein maturation process.

Apo-hSOD1 has been recently linked to the familial form of amyotrophic lateral sclerosis (fALS), a fatal motor neurodegenerative disease [8]–[10]. The immature form of hSOD1, i.e. without the metal ions and with a misfolded structure, is believed to play a pivotal role in ALS pathology [11], [12]. Therefore folding and metal insertion are important factors to be investigated in the cellular environment.

In this work, we characterized the state of hSOD1, analyzing samples of *E. coli* cells overexpressing hSOD1 protein, in its initial state after expression, and its initial maturation steps through zinc uptake and disulfide bond formation and we determined how this affects the tertiary and quaternary structure of the protein. This study sheds some light on the folding state of the non-mature protein as well as on the process of zinc uptake and protein folding in the cellular environment.

## Results

### Folding state of apo-hSOD1 in the cytoplasm

**Figure 1:**
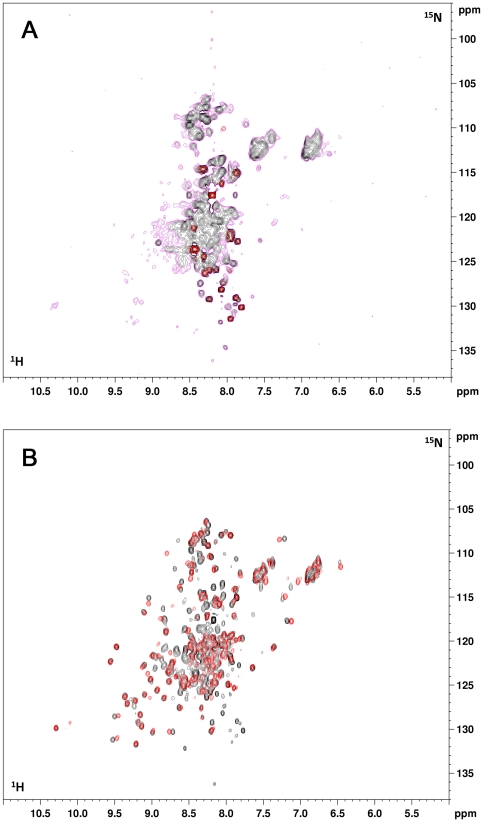
In-cell NMR spectra of apo-hSOD1 and of cell lysate. (A) In-cell ^1^H-^15^N SOFAST-HMQC spectrum of *E. coli* cells expressing hSOD1 in defect of Zn(II). Two different thresholds are shown (black, purple). At lower threshold (purple) some weak and broad signals are visible, while only signals in the unfolded region of the spectrum are visible at higher threshold (black). Signals belonging to other cellular components are overlaid in red. These are always present in all in-cell spectra. (B) Overlay of the ^1^H-^15^N SOFAST-HMQC spectrum of a cell lysate without addition of Zn(II) (black), and the ^1^H-^15^N HSQC of an *in vitro* sample of E,E-hSOD1^SH-SH^ (red).

^1^H,^15^N-SOFAST-HMQC spectra [13] were acquired on cell samples overexpressing hSOD1 in a metal-free medium. The spectra were then recorded again on the cleared lysates after cell lysis. The in-cell NMR spectrum shows mainly peaks in the 8.0–8.3 ppm (^1^H) region. In addition, few more dispersed peaks are visible, at lower S/N ratio (Figure 1 A). When the cells are lysed, still maintaining the sample in anaerobic conditions, a few other dispersed peaks appear, indicating the presence of some structured regions of the protein, while most of the peaks are still in the “unfolded” region. The spectrum of the latter species compares well with that of the monomeric apo form with reduced cysteines, E,E-hSOD1^SH-SH^(Figure 1 B) which also shows many peaks in the region of unstructured peptides, overall corresponding to the ones that remain visible in the in-cell spectrum of the protein. This therefore indicates that, once the newly produced protein is in the cytoplasm and in the absence of metal ions, it is in a reduced, metal-free state.

Several interpretations of the in-cell NMR spectrum of apo-hSOD1 are possible: apo-hSOD1 could be completely unfolded in the cytoplasm; in this case all the NH cross-peaks would be overlapped in the “unfolded” region of the spectrum. The protein could be only partially unfolded, as is the case of *in vitro* E,E-hSOD1^SH-SH^ [14], and the cross-peaks of the folded region could be lost as a consequence of chemical or conformational exchange phenomena in the NMR timescale. Alternatively, the loss of the signals could be a consequence of interactions with other components of the cellular environment, like membranes, bacterial heat shock proteins or DNA. Finally, the in-cell NMR species could be an oligomer of apo-hSOD1.

**Figure 2:**
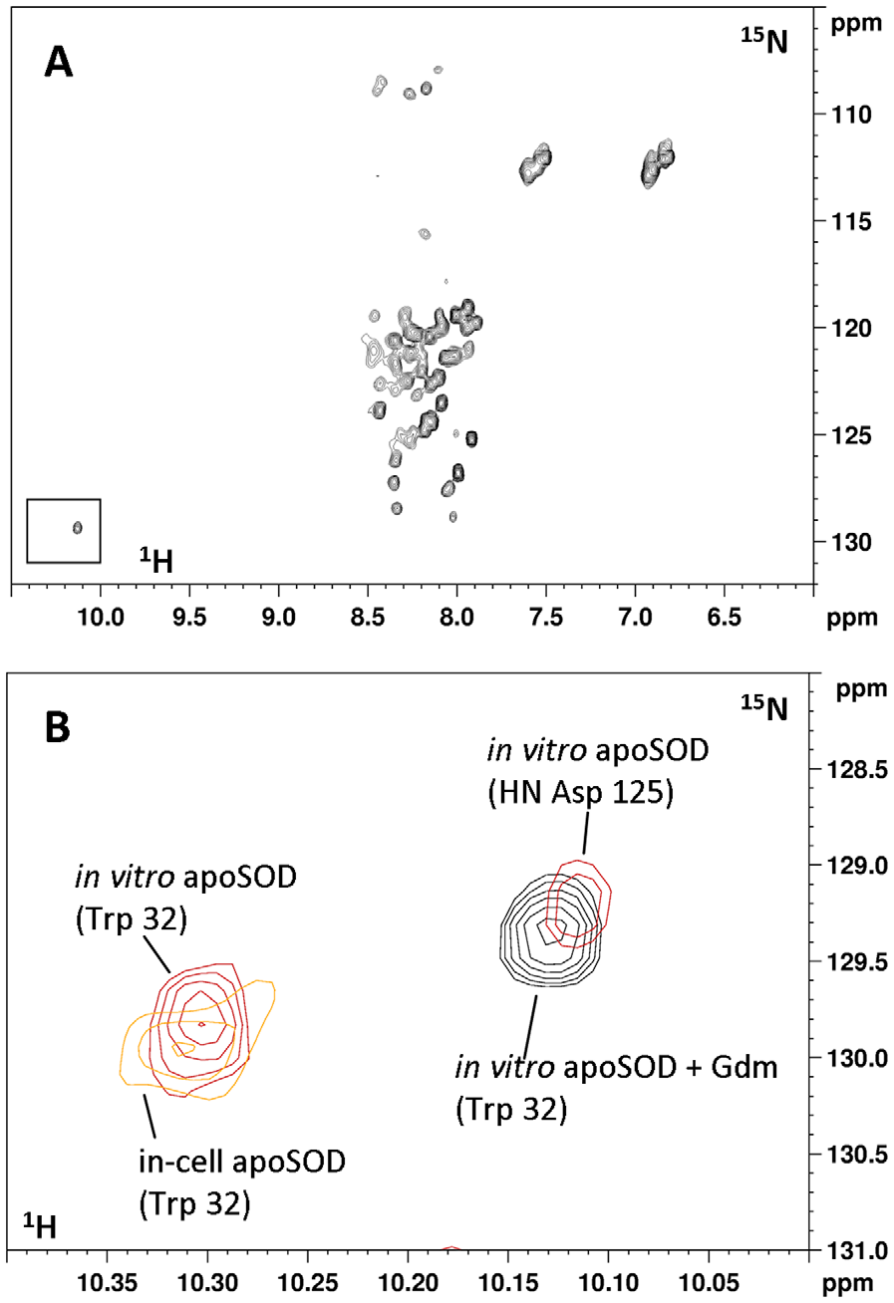
Trp 32 side chain of denatured *in vitro* apo-hSOD1. (A) ^1^H-^15^N HSQC of an *in vitro* sample of E,E-hSOD1^SH-SH^ denatured with 0.5 M guanidinium chloride. (B) Zoom of the (A) spectrum showing the signal of Trp 32 (black). Overlaid are the in-cell NMR spectrum of apo-hSOD1 (orange) and the *in vitro* spectrum of non-denatured E,E-hSOD1^SH-SH^.

To determine whether apo-hSOD1 is completely unfolded in the cytoplasm, the in-cell NMR spectrum was compared to the *in vitro* NMR spectrum of E,E-hSOD1^SH-SH^ denatured with guanidinium chloride (Figure 2 A). The latter spectrum also shows only signals in the region typical of unfolded proteins, but is somewhat different from the in-cell NMR spectrum. The cross peak of the side-chain NH of Trp 32 for example, which in the folded protein is located in a β-strand of the hSOD1 β-barrel, falls at different chemical shifts in the two spectra (Figure 2 B) and in particular in the cytoplasmic species it has the same chemical shift as in the *in vitro* E,E-hSOD1^SH-SH^. This indicates that in the cellular environment apo-hSOD1 is not completely unfolded, but the signals of the folded part are lost.

The loss of those signals could be caused by chemical or conformational exchanges phenomena involving the protein amides. In this case, the low S/N ratio of the signals should be increased at different magnetic field strength and temperature. SOFAST-HMQC spectra of cell samples expressing apo-hSOD1 were acquired at 500 MHz at 315 K and at 800 MHz at 288 K and 298 K. In both cases no increase of the S/N ratio of the signals was observed (data not shown), disfavouring this hypothesis.

The possibility of apo-hSOD1 forming aggregates in the cells was also taken into account. Apo-hSOD1 is known to give rise, both *in vivo* and *in vitro*, to soluble oligomeric species, and eventually to fibrils [11], [15]. It has been shown that the formation of soluble oligomers of apo-hSOD1 *in vitro* leads to the complete loss of the signals in the HSQC spectrum. This is most true for fibrils, which are insoluble species. Therefore, the presence of several signals in the “unfolded” region of the in-cell NMR spectrum, and the fact that the missing signals are completely recovered after cell lysis, excludes the possibility that oligomers of apo-hSOD1 have formed in the cytoplasm.

Apo-hSOD1 in the cytoplasm could be interacting to some extent with cellular components. Such interactions would lead to the formation of high molecular weight complexes, determining the broadening beyond detection of the signals. In this situation, only the amide signals of the unstructured regions are observable, as they might not interact with the cellular components and therefore move freely with respect to the slow-tumbling complex. Upon cell lysis these interactions are released, the protein becomes free and tumbles faster. The interactions have to be weak enough, in order to be disrupted upon sonication and subsequent dilution of the cell content in M9 buffer (∼1∶2 dilution).

The line broadening effect could be better explained considering the multiplicity of weak interactions in which apo-hSOD1 is involved. hSOD1 is overexpressed, and is more abundant than any other cellular species (∼850 µM in the cytoplasm). Therefore all the different interactions involve some hSOD1 molecules at any given time. In fact we can think of many sub-populations of apo-hSOD1, each experiencing a different chemical environment, thus having different chemical shifts. This “cellular anisotropy” causes an inhomogeneous broadening of different amount for each NH cross-peak. The peaks of the folded part have larger chemical shift dispersion and are thus made invisible by the cellular anisotropy.

### Zinc binding properties of cytoplasmic hSOD1

**Figure 3:**
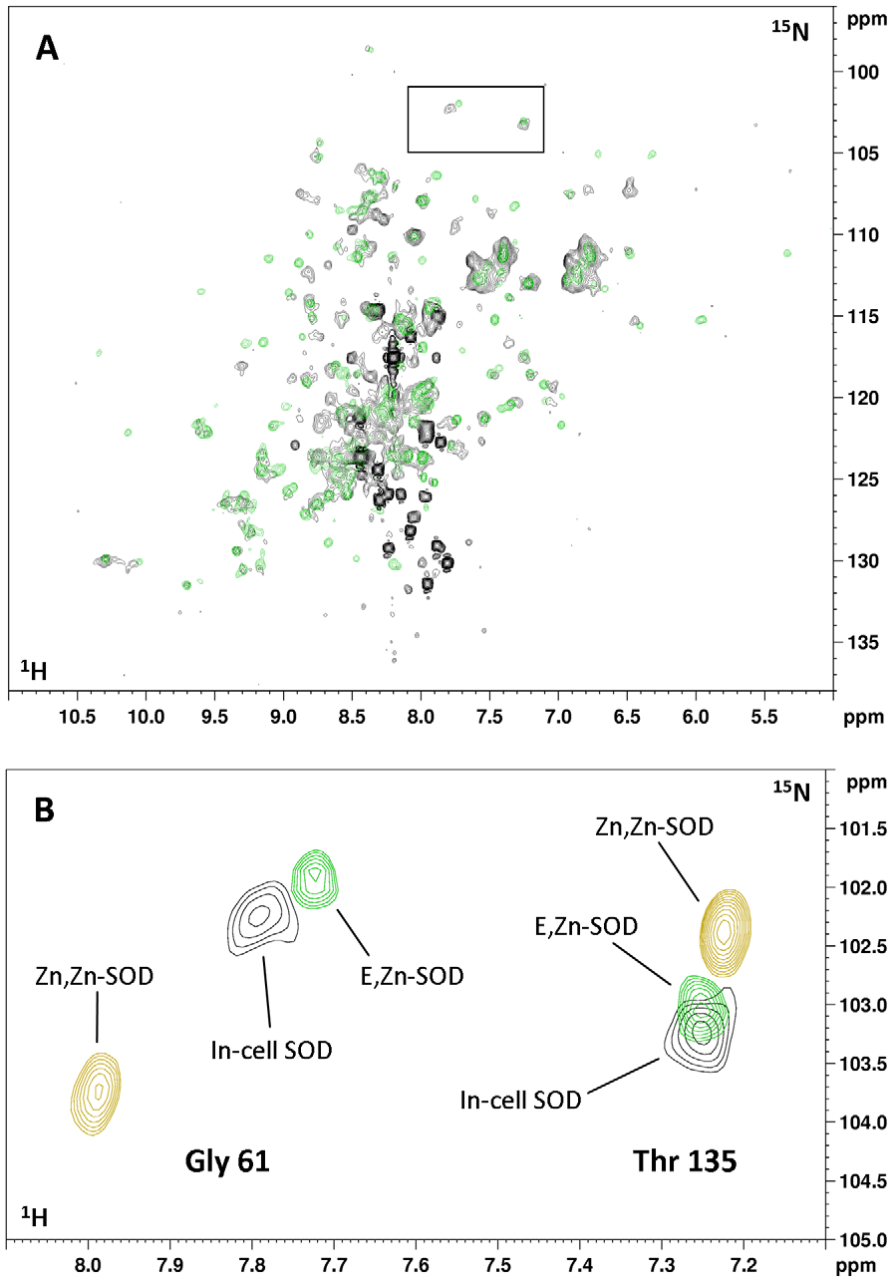
In-cell NMR spectra of hSOD1 with added Zn(II). (A) Overlay of the in-cell ^1^H-^15^N SOFAST-HMQC spectrum of *E. coli* cells expressing hSOD1 in presence of Zn(II) (black), and 1H-15N HSQC spectrum of an *in vitro* sample of E,Zn-hSOD1^S-S^ (green). (B) Zoom of the (A) spectrum showing the peaks of Gly 61 and Thr 135 in-cell (black), *in vitro* E,Zn-hSOD1^S-S^ (green) and *in vitro* Zn,Zn-hSOD1^S-S^ (yellow).

^1^H,^15^N-SOFAST-HMQC spectra were acquired on cell samples overexpressing hSOD1 in a minimal medium supplied with different amounts of ZnSO_4_. The concentration of zinc in the medium ranged from 10 µM to 1 mM in the expression medium; this is always in excess with respect to the total amount of hSOD1 expressed. When cells are grown in the presence of extra zinc added in the expression medium, the in-cell NMR spectra of hSOD1 show remarkable differences with respect to those of in-cell hSOD1 expressed without added zinc (Figure 3 A). The appearance of several dispersed peaks, together with the disappearance (or decrease in intensity) of some signals in the ‘unfolded’ region, indicates that hSOD1 inside the bacterial cells binds zinc when this is added to the culture medium in excess relatively to the total amount of protein expressed. This in-cell NMR spectrum compares very well with that of E,Zn-hSOD1^SH-SH^ (the species with one zinc ion bound to the zinc binding site), and not with that of Zn,Zn-hSOD1^SH-SH^ (the non-physiological species with two zinc ions bound to both metal binding sites). Among the various signals, the NH signals from Gly 61 and Thr 135, which are close to the metal binding sites, have chemical shift values which are indicative of the metal binding state of the protein, i.e. of which metal site is occupied by which metal ion. In the in-cell NMR spectra these signals have chemical shifts very close to those observed in the E,Zn-hSOD1^SH-SH^ *in vitro* spectrum (combined chemical shift difference ^1^H-^15^N: ΔδG61 = 0.015, ΔδT135 = 0.029), while are more distant from the corresponding signals of the Zn,Zn-hSOD1^SH-SH^ form (ΔδG61 = 0.169, ΔδδT135 = 0.123) (Figure 3 B, Figure S1). This is a striking result as it indicates that hSOD1 in the cytoplasm has a higher selectivity than *in vitro* in the binding site mode. Indeed, when sub-stoichiometric amounts up to 1 equivalent of zinc are added to E,E-hSOD1 *in vitro* at physiological conditions (pH around 7), mixtures of E,E-hSOD1, E,Zn-hSOD1 and Zn,Zn-hSOD1 species are formed, while when 2 equivalents of zinc per subunit are added only Zn,Zn-hSOD1 is formed. Instead in the cytoplasm hSOD1 binds zinc only in its native binding site, giving only E,Zn-hSOD1 species, while zinc binding to the copper site does not occur, and Zn,Zn-hSOD1 species is not detected (within the sensitivity of the NMR experiment). Moreover, this effect is seen regardless of the concentration of zinc added to the medium, even at 1 mM.

After the in-cell NMR experiments, the cell samples were washed with metal-free medium in order to remove the external zinc, and NMR spectra of the cleared cell lysates were acquired. Only the species Zn,Zn-hSOD1^SH-SH^ was detected. Apparently, an excess of zinc is still present inside the cells, which is made available upon cell lysis and binds at the hSOD1 copper binding site.

### Cysteine redox state determination

hSOD1, when is either in the cytoplasm or in the cell lysate, without addition of zinc has all its cysteines in the reduced state, as monitored from ^1^H-^15^N signals of selectively labelled cysteines.

**Figure 4:**
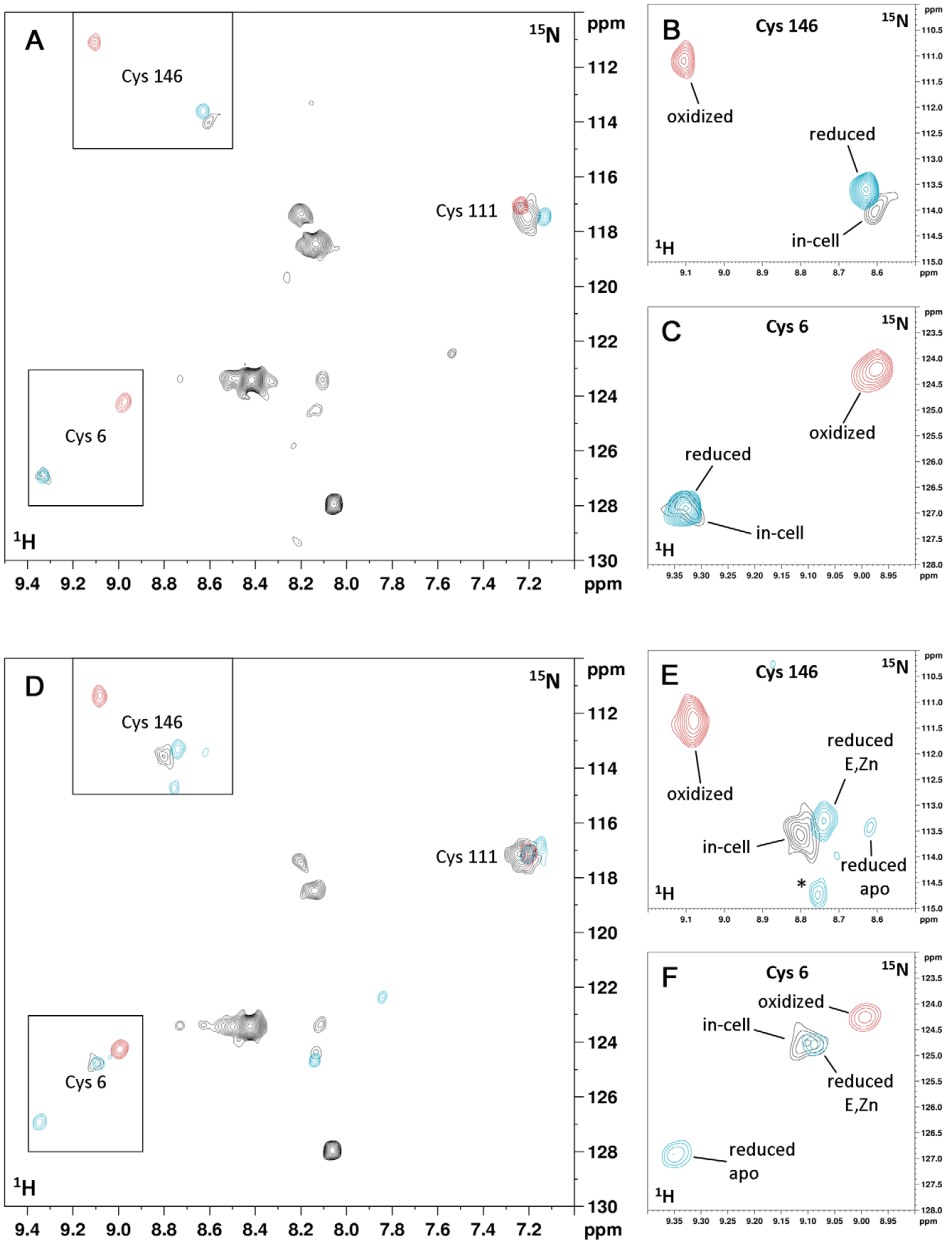
In-cell and *in vitro* NMR spectra of ^15^N cysteine-labelled E,E-hSOD1 and E,Zn-hSOD1. (A) Overlay of ^1^H-^15^N SOFAST-HMQC in-cell spectrum (black) of ^15^N cysteine-labelled E,E-hSOD1, oxidized E,E-hSOD1 purified from the lysate (red), same sample reduced with DTT (blue). (B,C) Detailed views of (A) showing Cys 146 and Cys 6 amide cross-peaks. (D) Overlay of ^1^H-^15^N SOFAST-HMQC in-cell spectrum (black) of ^15^N cysteine-labelled E,Zn-hSOD1, oxidized E,Zn-hSOD1 purified from the lysate (red), same sample reduced with DTT (blue). (E,F) Detailed views of (D) showing Cys 146 and Cys 6 amide cross-peaks. When DTT is added to the sample of E,Zn-hSOD1^S-S^, E,Zn-hSOD1^SH-SH^ is detected, together with E,E-hSOD1^SH-SH^ and other species (indicated with an asterisk).

After cysteine oxidation by air exposure, the spectrum shows the four peaks of the cysteines, with that of Cys 57 only detectable below 298K. Cys 57 and 146 have chemical shifts equal to those previously assigned in the *in vitro* dimeric E,E-hSOD1^S-S^ species [16] and indicative of their oxidized state. After reduction of the same sample with DTT, the peaks shift back to the chemical shift they have in the cells and in the cell lysate, therefore confirming the reduced state of Cys 57 and 146 in the cytoplasm (Figure 4 A–C). The same analysis, repeated for cells and lysates when hSOD1 is expressed in the presence of Zn(II), shows that also in the case of E,Zn-hSOD1, Cys 57 and 146 in the cytoplasm are reduced (Figure 4 D–F).

The redox state of the cysteines is further confirmed by reaction with AMS performed directly on the cell culture and on the cell lysate after the NMR experiments (Figure S2, Information S1).

### Quaternary structure of hSOD1

The spectra of the ^15^N-cysteine labelled protein provide evidences on the quaternary structure of hSOD1 in the cellular cytoplasm. Indeed disulfide bond formation and protein dimerization are tightly linked processes. *In vitro*, the species E,E-hSOD1^SH-SH^ is monomeric, while both E,E-hSOD1^S-S^ and E,Zn-hSOD1^SH-SH^ are homodimers [14], [17]. In the NMR spectra of both *in vitro* ^15^N-cysteine labelled E,E-hSOD1 and E,Zn-hSOD1, the amide cross-peak of Cys 146 has a large chemical shift difference between the two redox states (Figure 4 B,D). This difference is expected, as Cys 146 is directly involved in the disulfide formation. On the other hand, the cross-peak of Cys 6 has a similar behaviour upon disulfide formation in E,E-hSOD1, but changes little in the case of E,Zn-hSOD1 (Figure 4 C,F). This is because Cys 6 is located close to the interaction surface of the homodimer. Upon E,E-hSOD1 dimerization as a consequence of disulfide bond formation, Cys 6 changes its chemical environment, whereas in E,Zn-hSOD1, which is always dimeric regardless of the oxidation state, it remains unchanged. Therefore the chemical shift of Cys 6 is a marker of the quaternary structure of hSOD1. The chemical shift of Cys 6 of both in-cell E,E-hSOD1^SH-SH^ and E,Zn-hSOD1^SH-SH^ species matches that of the corresponding *in vitro* species, thus suggesting that in the cytoplasm E,E-hSOD1^SH-SH^ is in the monomeric state, while E,Zn-hSOD1^SH-SH^ is in the dimeric state, confirming that the *in vitro* findings hold true also inside the cell.

## Discussion

The process leading a newly synthesized protein to acquire its final, functional state could involve several steps which consist of protein folding to its tertiary and possibly quaternary structure, cofactor binding and post-translational modifications. In most of the cases these steps have been characterized at molecular level only *in vitro*, where protein is isolated in an environment which might be far from the physiological one. In-cell NMR is a quite powerful method to characterize in detail those processes directly in living cells. Indeed, the atomic resolution of the technique, which can be applied to labelled proteins while inside living cells, allows us not only to analyze the folding and organization properties of a protein but also to look at the status of individual residues and the interaction with cofactors.

hSOD1 needs to undergo a number of events after its synthesis, i.e. needs to bind one zinc and one copper ion per molecule, to form a disulfide bond and to dimerize. In this work we have analyzed the protein state directly in the cytoplasm of *E. coli* cells, before it undergoes those maturation steps. Then, by adding zinc to the external medium, we have been able to monitor the changes in tertiary and quaternary structure following the binding of zinc. Additionally, we have shown that during this process the cysteines involved in the intramolecular disulfide bridge are in the reduced state. Structural information was obtained by comparing the in-cell NMR spectra with spectra recorded *in vitro* on single, defined protein states.

We have shown that apo-hSOD1 in the cytoplasm is monomeric, reduced and in a partially unstructured state. This is the species which has been suggested and is believed to pass the outer membrane of mitochondria, reaching the IMS where it is trapped upon interaction with the copper chaperone for SOD (CCS) [18]. Interestingly, apo-hSOD1 in the *E. coli* cytoplasm appears to be interacting with different cellular components, giving rise to a distribution of conformations which causes the broadening of part of the NH signals.

A striking result of the present characterization is the in cell selectivity for zinc binding. We have shown that when zinc is added in excess to the culture medium, hSOD1 binds only one equivalent of zinc in its native site, while *in vitro* both metal binding sites are able to bind zinc with comparable affinity. The selective binding of only one zinc ion per subunit occurs only in the intact cells, as when they are lysed and hSOD1 is exposed to an excess of zinc, it binds two equivalents per subunit. Zinc uptake is known to be tightly regulated in the cytoplasm of *E. coli*. While the concentration of free zinc is reported to be less than one atom per cell, the total amount of zinc present is thought to be distributed among other cellular species, either zinc binding proteins or small molecules [19]. It is therefore possible that in this regulated environment zinc binding to hSOD1 becomes highly selective towards the zinc binding site, contrarily to what occurs *in vitro*. Conversely, when the cells are lysed the zinc distribution is not controlled anymore and, if zinc in excess is present, Zn,Zn-hSOD1 species is formed.

To reach its mature state, E,Zn-hSOD1^SH-SH^ still has to bind a copper ion per monomer and the disulfide bridge has to form. However, in most of Gram-negative bacteria, such as *E. coli*, no copper proteins are known to localize in the cytoplasm, and indeed these organisms have copper efflux pumps to remove copper from the cytoplasm, while no protein is present for cytoplasmic copper uptake [20]. Therefore hSOD1 cannot bind copper after zinc, even if copper is added to the culture medium. Copper binding of course can occur in the cytoplasm of eukaryotic cells, which have systems to regulate cytoplasmic copper intake [21]–[23], provided the CCS chaperone is present.

Concerning the cysteine redox state, we have shown that after zinc binding the formation of the disulfide bridge still does not occur. This suggests that in the eukaryotic cells the disulfide formation process is also mediated by CCS, and it may be concomitant to the copper loading.

## Methods

### Cell samples preparation

For uniform ^15^N labelling, BL21(DE3) Gold *E. coli* strain was used, transformed with a pET28a plasmid containing the WT hSOD1 gene sequence without any additional tag. For selective ^15^N-cysteine labelling, the auxotroph strain BL21(DE3) CysE was transformed with a pET21 plasmid containing the same WT hSOD1 gene sequence.

Cell samples for in-cell NMR were prepared by adapting a reported protocol [24] (Information S2).

For expression of selective ^15^N-cysteine labelled WT hSOD1 the samples were prepared as described above, but a M9-based reconstituted medium was used [25], containing ^15^N-cysteine and the other 19 unlabelled amino acids.

Concentration of hSOD1 inside the cytoplasm after 4 h of overexpression was determined by measuring the absorption of the hSOD1 band on a coomassie-stained SDS-PAGE. A purified hSOD1 sample of known concentration (200 uM) was run on the same gel at different dilutions to provide a calibration curve. hSOD1 is ∼850 µM in the cytoplasm, while in the lysate sample hSOD1 is ∼350 uM.

### Purified hSOD1 samples preparation

Pure WT hSOD1 protein was prepared following an existing protocol [26] (Information S3).

The removal of the metals to obtain E,E-hSOD1^S-S^ was achieved by dialyzing several times a diluted solution of hSOD1 against 10 mM EDTA in 50 mM acetic acid at pH 3.5. After removal of EDTA E,Zn-hSOD1^S-S^ and Zn,Zn-hSOD1^S-S^ were then obtained by adding at pH 5.5 one and two equivalents of ZnSO_4_, respectively.

To obtain E,E-hSOD1^SH-SH^ and E,Zn-hSOD1^SH-SH^, the E,E-hSOD1^S-S^ and E,Zn-hSOD1^S-S^ were incubated 1 h at 37°C with 50–60 mM of DTT; 1 mM EDTA was added to the sample of E,E-hSOD1^SH-SH^ to prevent binding of any metal present in traces. DTT concentration was then brought to 2 mM by dialysis against oxygen-free phosphate buffer. Zn,Zn-hSOD1^SH-SH^ was obtained by adding ZnSO_4_ in excess (4 equivalents) to a sample of reduced E,E-hSOD1^SH-SH^.

The final NMR samples obtained were in 20 mM phosphate buffer at pH 7.5; protein concentration ranged between 0.1 and 0.3 mM (referred to the monomer). All NMR spectra were acquired at Bruker Biospin 600 and 800 MHz spectrometers. The latter is equipped with a cryo-cooled TXI probe.

### In-cell NMR experiments

We performed a series of in-cell NMR experiments in which protein expression was induced in M9 medium either without zinc or with increasing amounts of ZnSO_4_ (from 10 µM up to 1 mM). 2D ^1^H,^15^N-SOFAST-HMQC spectra were acquired at 310K. The total acquisition time for each cell sample ranged from 1 to 3 h. After the acquisition, the cells were gently centrifuged and collected to be lysed. The supernatant was then checked in the same experimental conditions, in order to exclude the presence of any signal arising from the protein leaked out of the cells (**Figure S3**). After cell lysis by sonication and centrifugation, 2D ^1^H,^15^N-SOFAST-HMQC spectra were acquired on the cleared cell lysate.

The cysteine redox state inside the cells and in the cell lysate was determined via NMR by monitoring the ^1^H and ^15^N chemical shifts of the ^15^N-labelled cysteines. After lysis of the in-cell NMR sample, the protein contained was roughly purified with DEAE anion exchange resin, and left exposed to air for >2 h to allow cysteine oxidation. Finally, *in vitro* reduction of the cysteines was performed with 50 mM DTT.

### In vitro NMR experiments

Either 2D ^1^H,^15^N-HSQC spectra or both ^1^H,^15^N-HSQC and ^1^H,^15^N-SOFAST-HMQC spectra were acquired on the *in vitro* ^15^N-labelled samples of WT hSOD1 at 310K. The linewidth of the HSQC crosspeaks is 10% smaller on average than that of the SOFAST-HMQC crosspeaks, while the crosspeak intensities are comparable, the chemical shifts being the same. Unfolding of an *in vitro* sample of E,E-hSOD1^SH-SH^ was performed by adding increasing amounts of guanidinium chloride up to 0.5 M, and was monitored with 2D ^1^H,^15^N-HSQC spectra acquired at 600 MHz. ^15^N R_1_ and R_2_ relaxation measurements [27] were performed at 600 MHz on the ^15^N labelled E,E-hSOD1^SH-SH^ sample. The τ_m_ estimated from R_2_/R_1_ ratios (τ_m_ = 11.1±1.5 µs) confirmed that the protein was in the monomeric state [14].

## Supporting Information

Figure S1
**Combined Chemical Shift Difference (CCSD) plot of in-cell vs.** ***in vitro*** **zinc-bound hSOD1 NMR spectra.** CCSD plot of a subset of amide resonances of hSOD1 showing that the in-cell+zinc hSOD1 species is more similar to *in vitro* E,Zn-hSOD^SH-SH^ compared to *in vitro* Zn,Zn-hSOD1^SH-SH^. CCSDs between in-cell+zinc hSOD1 and Zn,Zn-hSOD^SH-SH^ (blue) are higher on average than CCSDs between in-cell+zinc hSOD1 and E,Zn-hSOD^SH-SH^ (orange). Amide cross-peaks of residues Gly 61 and Thr 135 (marked with an asterisk) are shown in **Figure 3B**. CCSDs were calculated using the formula: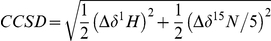.(TIF)

Figure S2 **Cysteine redox state determined by reaction of hSOD1 with AMS.** Non-reducing SDS-PAGE of AMS reaction performed on cell cultures expressing hSOD1 both in presence and in defect of Zn(II) in the medium (right). AMS reaction on *in vitro* samples (left) of reduced and oxidized hSOD1 is showed as a reference.
(TIF)

Figure S3 **Supernatant after centrifugation of the cell sample.** ^1^H-^15^N SOFAST-HMQC spectrum of the supernatant collected after centrifugation of an in-cell NMR sample of hSOD1. The threshold has been lowered to show the very low S/N ratio of the signals detected.
(TIF)

Information S1 **Reaction with AMS.**
(DOC)

Information S2 **Cell and lysate samples preparation.**
(DOC)

Information S3 **hSOD1 purification protocol.**
(DOC)
