# Supplementary figures and images for: In-cell NMR in E. coli to Monitor Maturation Steps of hSOD1
Source: PLoS One. 2011 Aug 24;6(8):e23561. doi: 10.1371/journal.pone.0023561 (PMC3160886; doi:10.1371/journal.pone.0023561)

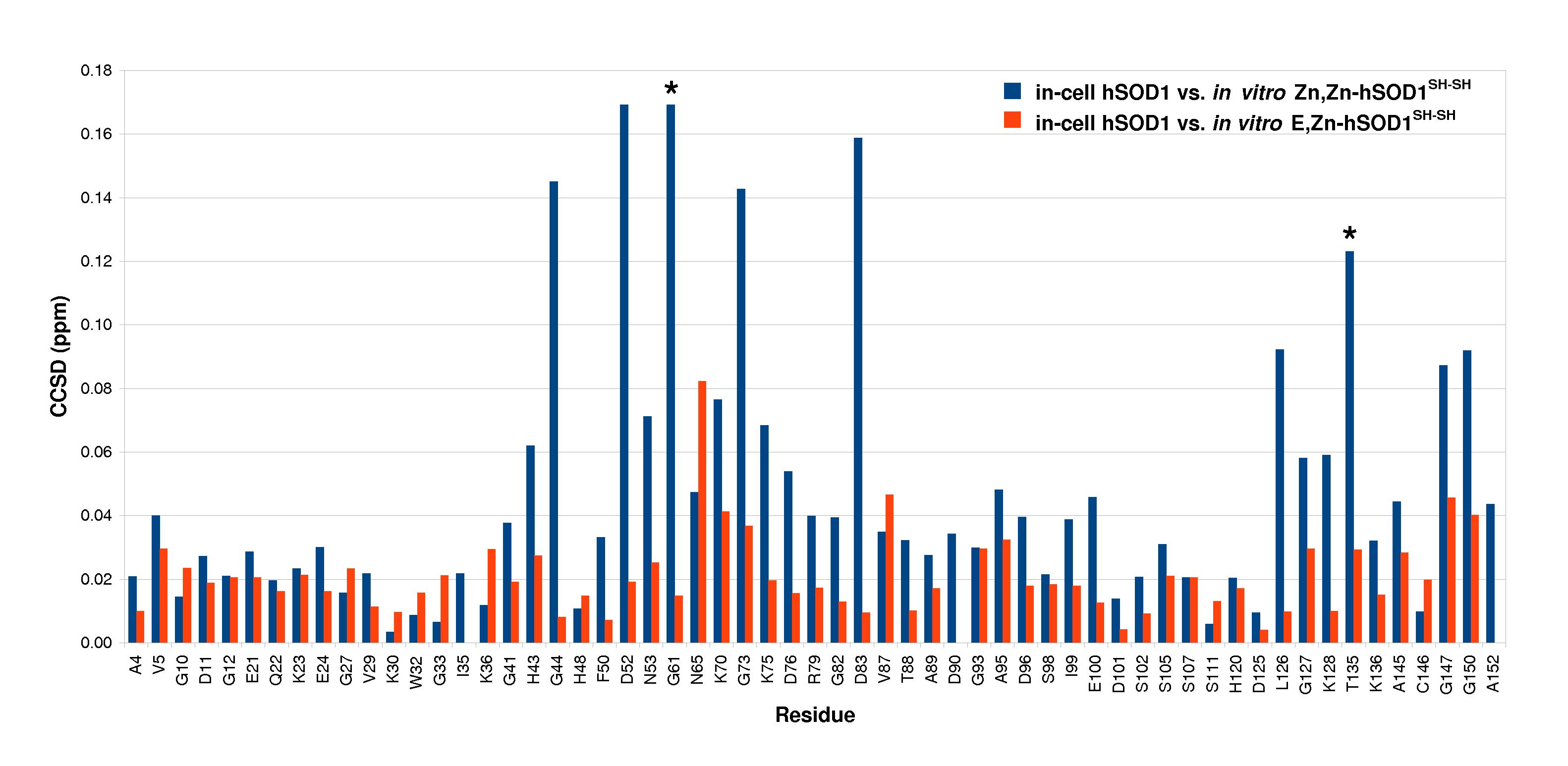

Supplement: Figure S1 — Combined Chemical Shift Difference (CCSD) plot of in-cell vs. in vitro zinc-bound hSOD1 NMR spectra. CCSD plot of a subset of amide resonances of hSOD1 showing that the in-cell+zinc hSOD1 species is more similar to in vitro E,Zn-hSODSH-SH compared to in vitro Zn,Zn-hSOD1SH-SH. CCSDs between in-cell+zinc hSOD1 and Zn,Zn-hSODSH-SH (blue) are higher on average than CCSDs between in-cell+zinc hSOD1 and E,Zn-hSODSH-SH (orange). Amide cross-peaks of residues Gly 61 and Thr 135 (marked with an asterisk) are shown in Figure 3B . CCSDs were calculated using the formula:. (TIF) [file pone.0023561.s001.tif]

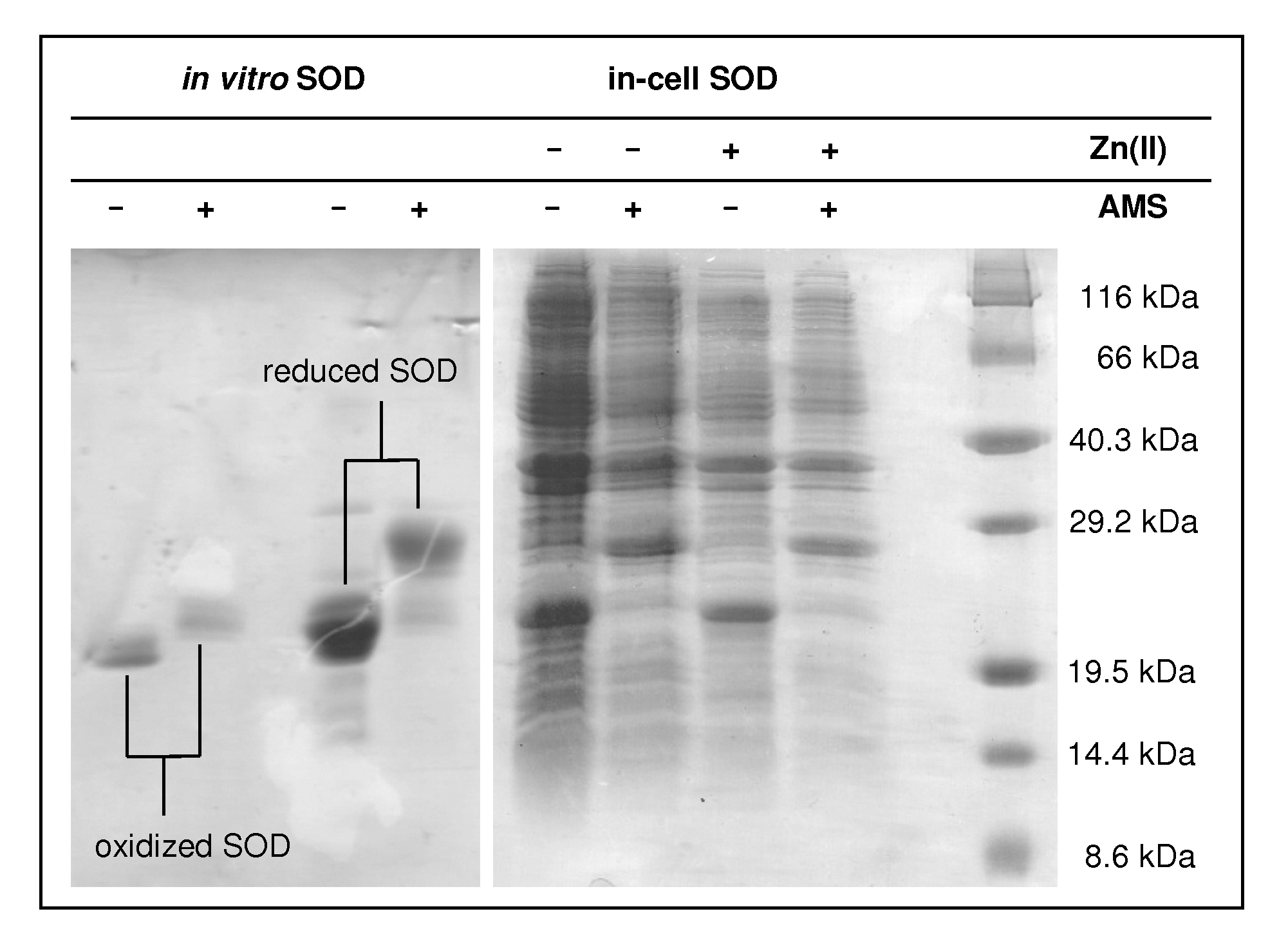

Supplement: Figure S2 — Cysteine redox state determined by reaction of hSOD1 with AMS. Non-reducing SDS-PAGE of AMS reaction performed on cell cultures expressing hSOD1 both in presence and in defect of Zn(II) in the medium (right). AMS reaction on in vitro samples (left) of reduced and oxidized hSOD1 is showed as a reference. (TIF) [file pone.0023561.s002.tif]

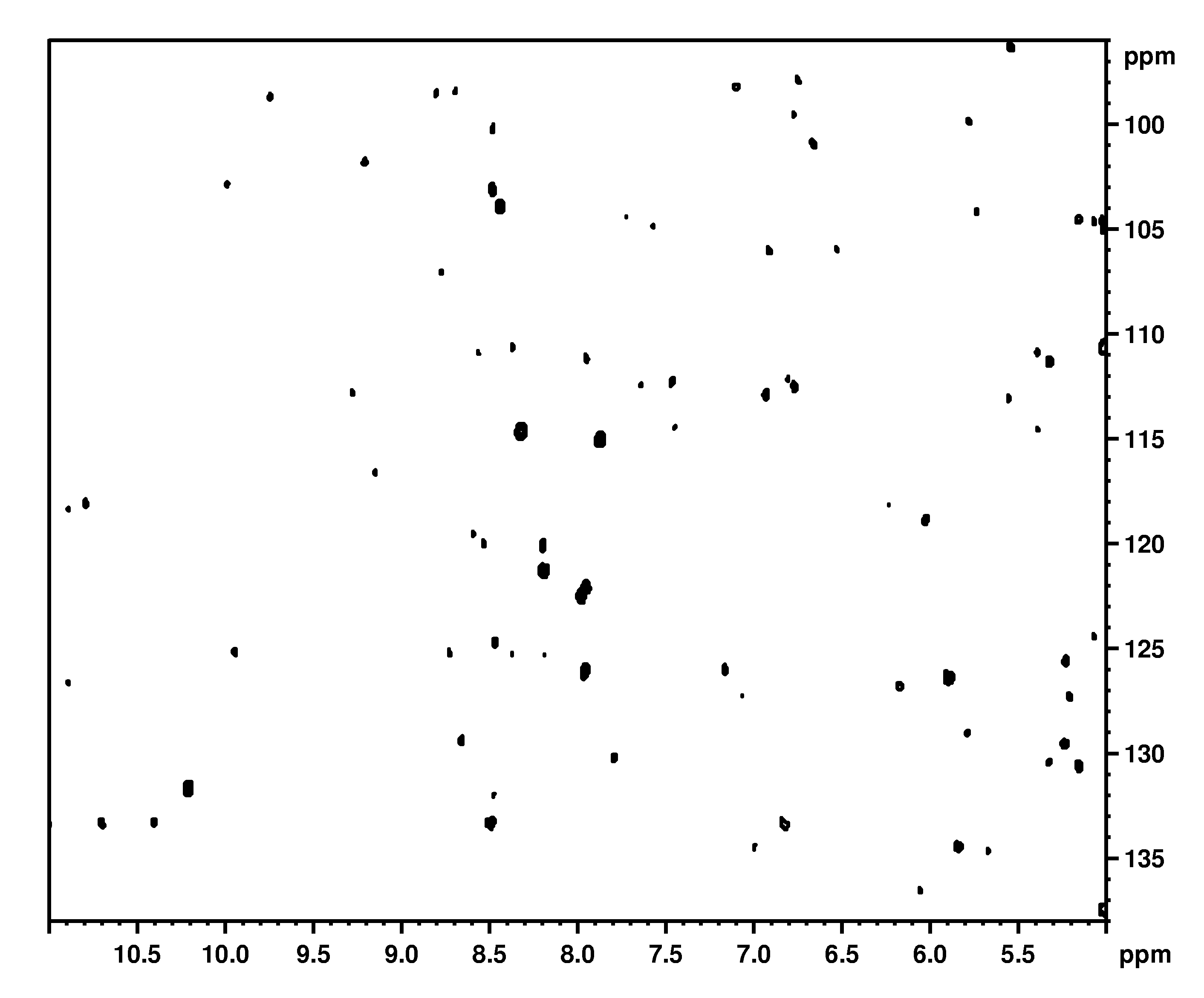

Supplement: Figure S3 — Supernatant after centrifugation of the cell sample. 1H-15N SOFAST-HMQC spectrum of the supernatant collected after centrifugation of an in-cell NMR sample of hSOD1. The threshold has been lowered to show the very low S/N ratio of the signals detected. (TIF) [file pone.0023561.s003.tif]
