# Supplementary material for: In-cell NMR in E. coli to Monitor Maturation Steps of hSOD1
Source: PLoS One. 2011 Aug 24;6(8):e23561. doi: 10.1371/journal.pone.0023561 (PMC3160886; doi:10.1371/journal.pone.0023561)
Supplement: Information S1 — Reaction with AMS. (DOC) [file pone.0023561.s004.doc]

**Supporting Information S1. Reaction with AMS**

Reaction with 4-acetamido-4’-maleimidylstilbene-2,2’-disulfonic acid (AMS) was performed directly on cell samples in oxygen-free conditions [1]. 1 mL of cell culture was precipitated with 10% trichloroacetic acid (TCA), washed with 80 µL acetone and re-suspended in 100 µL 100 mM Tris pH 7 + 2% SDS. 10 µL of the mixture obtained was incubated 1 h at 37°C with 20 mM AMS, and finally run on a non-reducing SDS-PAGE. The same reaction was performed on *in vitro* samples of hSOD1^S-S^ and hSOD1^SH-SH^. 20 µL of an *in vitro* protein sample (0.2 mM) were precipitated with 10% TCA, washed with 40 µL acetone and re-suspended in 50 µL 100 mM Tris pH 7 + 2% SDS. 10 µL of the mixture obtained was incubated 1 h at 37°C with 20 mM AMS.

References

1. Kobayashi T, Ito K (1999) Respiratory chain strongly oxidizes the CXXC motif of DsbB in the Escherichia coli disulfide bond formation pathway. EMBO J. 18: 1192-1198.
