# Supplementary material for: In-cell NMR in E. coli to Monitor Maturation Steps of hSOD1
Source: PLoS One. 2011 Aug 24;6(8):e23561. doi: 10.1371/journal.pone.0023561 (PMC3160886; doi:10.1371/journal.pone.0023561)
Supplement: Information S2 — Cell and lysate samples preparation. (DOC) [file pone.0023561.s005.doc]

**Supporting Information S2. Cell and lysate samples preparation**

*Cell samples preparation*

The M9 buffer used in all cell cultures was checked through ICP-AES for traces of zinc. The concentration of Zn^2+^ was below the detection limit of 0.04 µM.

Cell samples for in-cell NMR were prepared as follows: a cell culture was grown overnight at 30°C in 35 mL of LB medium. After gentle centrifugation (3000 g) for 20 minutes, the cells were re-suspended in 50 mL of M9 minimal medium [M9 buffer (7 g/L K_2_HPO_4_, 3 g/L KH_2_PO_4_, 0.5 g/L NaCl, pH 7.4), 2 mM MgSO_4_, 0.1 mM CaCl_2_, 1 mg/L biotin, 1 mg/L thiamine, antibiotic] containing 1 g/L (^15^NH_4_)_2_SO_4_ and 3 g/L of unlabelled glucose to obtain an OD_600_ of ~1.6. After 10 min recovery time, overexpression was induced with 0.5 mM IPTG, and carried out at 30°C for 4 h. The cells were washed once with 50 mL of metal-free M9 buffer in order to remove nutrients, metal ions and any excreted by-product, and they were harvested through gentle centrifugation. The pellet was then re-suspended in metal-free M9 buffer until 500 µL of a ~50% v./v. cell slurry were obtained. 50 µL of D_2_O were added, and the final volume was put in a 5 mm NMR tube.

*Cell lysates preparation*

Cleared cell lysates for in-cell NMR experiments were prepared as follows: after removal of the supernatant to be checked by NMR, the cell pellet was re-suspended in an equal volume of metal-free M9 buffer. The cells were then lysed by ultrasonication. Then the lysate was centrifuged at 18000 g for 20 minutes, the supernatant was collected and its volume was brought to 500 µL with M9 buffer. 50 µL of D_2_O were then added. The final dilution of the cytoplasm in M9 buffer is around 1:2.
