# Supplementary material for: In-cell NMR in E. coli to Monitor Maturation Steps of hSOD1
Source: PLoS One. 2011 Aug 24;6(8):e23561. doi: 10.1371/journal.pone.0023561 (PMC3160886; doi:10.1371/journal.pone.0023561)
Supplement: Information S3 — hSOD1 purification protocol. (DOC) [file pone.0023561.s006.doc]

**Supporting Information S3. hSOD1 purification protocol**

Pure hSOD1 protein was prepared as follows: a cell culture (BL21(DE3) Gold (Stratagene), transformed with a pET28a plasmid containing the WT hSOD1 gene) was grown overnight at 30°C in 750 mL LB, harvested and re-suspended in 2.25 L ^15^N-labelled M9 medium. After 4 h from induction with 0.5 mM IPTG at 30°C the cells were harvested and re-suspended in 20 mM Tris, pH 8 buffer for lysis. The cleared lysate was loaded on an anion exchange column (DEAE Sepharose Fast Flow resin, GE Healthcare) for a first purification of hSOD1 by elution with NaCl gradient. The collected fractions containing hSOD1 (checked by SDS-PAGE) were further purified by gel filtration (Superdex75 16/60 column, GE Healthcare) in 20 mM Tris, 100 mM NaCl, pH 8 buffer. Fractions containing pure hSOD1 were collected. ZnSO_4_ was added to the protein solution to increase hSOD1 stability, and 1 mM DTT was added in all buffers to prevent protein aggregation through disulfide bridges.
